# Variations of amino acid, free carnitine and acylarnitine profiles analysed by tandem mass spectrometry in newborns during their first four weeks of life

**DOI:** 10.1186/1687-9856-2015-S1-P120

**Published:** 2015-04-28

**Authors:** Fang Hong, Xinwen Huang, Zhenzhen Hu, Fan Tong, Rulai Yang, Zhengyan Zhao

**Affiliations:** 1 Department of Genetics and Metabolism, Children’s Hospital, Zhejiang University School of Medicine, Hangzhou, Zhejiang, China

## Aims

Electrospray ionization-tandem mass spectrometry (MS/MS) has increasingly been advocated for expanded newborn screening and for the early diagnosis of amino acidemias, fatty acid oxidation disorders, and organic acidurias. Here, we characterized amino acid, free carnitine, and acylcarnitine status with respect to postnatal ages in 158,730 newborns over the last two years.

## Methods

Six age groups were defined: 3-4 d (Group A), 5-7 d (Group B), 8-10 d (Group C), 11-15 d (Group D), 16-20 d (Group E), and 21-28 d (Group F). Amino acid, free carnitine, and acylcarnitine concentrations of dried whole blood spots were obtained from the MS/MS analysis. Linear regression analyses of all results of highly disease-related analytes were carried out to identify covariates associated with sampling time in Matlab. For the respective regression coefficients (RC), plus represents the analyte levels elevate as the sampling time prolongs, and vice versa for minus.

## Results

Except for PHE, ALA and GLY, values of the remaining amino acids increased with postnatal age as the regression coefficients were plus. In addition, most amino acids levels during days 5-10 were distinctly higher with the exceptions of ALA, ORN and GLY. For free carnitine and short-chain ACs, C0, C5 and C5OH elevated slightly with sampling time (RC<0.05), while analytes left decreased along with age especially for C2 and C3 (RC for C3 was 0.01). For medium- and long-chain ACs, all analytes concentrations decreased with specimen collection time. C14, C16, C18, and C18:1 turned out to remain only half of their primary levels; and the level of C16 varied from 2.73 μmol/L in Day 3-4 to 0.84 μmol/L in Day 21-28. Our results indicate that most amino acids and acylcarnitines had no obvious age-dependent varieties in concentration levels during neonatal period with exceptions of C2, C3, C14, C16, C18, C18:1 and C16.

## Conclusions

Although the risk of underdiagnosis of IEM with the use of the same newborn values as reference can be considered as small, for some parameters the use of age-related reference values may have a potential impact on the diagnosis and management of inherited errors of metabolism.

